# Bibliography

**Published:** 1890-04

**Authors:** 


### BIBLIOGRAPHY.

TRANSACTIONS OF THE AMERICAN DENTAL ASSOCIATION, at the Twenty-ninth Annual Session,—Held at Saratoga Springs, N. Y., August, 1889. Philadelphia: S. S. White Dental Manufacturing Company, 1889.

DENTAL CHEMISTRY AND METALLURGY. By CLIFFORD MITCHELL, M.D. Chicago: W. T. Keener, 1890.

This is the second edition of the Dentist's Manual of Special Chemistry, revised, rewritten, and including the following parts: 1. Essentials of Chemistry for Dental Students. 2. General Chemistry for Dental Practitioners. 3. Laboratory Course in Elementary Chemistry for Dental Students. 4. Laboratory Course in Dental Chemistry and Metallurgy.

The author rightly claims that a knowledge of chemistry is of the greatest value to the dental practitioner, and he predicts that, as the special requirements of the dentist are different from those of the medical man, in time the dental student will have a distinct course in this branch. The work is well written, the matter neatly arranged, and printed in good large type.

ARTIFICIAL CROWN- AND BRIDGE-WORK. By GEORGE EVANS. Second Edition, Revised and Enlarged. 547 illustrations. Philadelphia: S. S. White Dental Manufacturing Company, 1889.

The rapidity of growth of this kind of work, and the consequent changes it has undergone, may be judged by the fact that in less than a year after the publication of the first edition of his work the author has deemed a second necessary. It is fully up to date, and embracing, as it does, the methods invented by, and the practice in vogue among, the most advanced dentists, it must prove of much value to the progressive practitioner.

